# Protocols on classification, monitoring and therapy in children’s rheumatology (PRO-KIND): results of the working group Polyarticular juvenile idiopathic arthritis

**DOI:** 10.1186/s12969-017-0206-9

**Published:** 2017-11-07

**Authors:** Gerd Horneff, Ariane Klein, Gerd Ganser, Michaela Sailer-Höck, Annette Günther, Ivan Foeldvari, Frank Weller-Heinemann

**Affiliations:** 1 Department of Pediatrics, Asklepios Clinic Sankt Augustin, Arnold Janssen Str., 29, 53757 Sankt Augustin, Germany; 2 Department of Pediatric Rheumatology, St. Josef Hospital, Sendenhorst, Germany; 3 Department of Pediatrics I, Medical University of Innsbruck, Innsbruck, Austria; 4 Department of Pediatric Pulmonology and Immunology, Behring Hospital, Berlin, Germany; 5 Centre Pediatric Rheumatology, Hamburg, Germany; 6 Department of Pediatrics, Prof. Hess Children’s Hospital, Bremen, Germany

**Keywords:** Polyarticular juvenile idiopathic arthritis, Treat to target, Treatment

## Abstract

**Objective:**

Several effective pharmacologic treatment options for polyarticual juvenile idiopathic arthritis (JIA) have emerged but initial treatment is heterogeneous in Germany. Therefore, the German Society of Pediatric Rheumatolgy has established a commission to develop consensus “Protocols on classification, monitoring and therapy in children's rheumatology (PRO-KIND)” to harmonize diagnostic and treatment approaches for new-onset JIA in Germany.

**Methods:**

A set of definitions for in- and exclusion, diagnostic workup, parameters for the evaluation of disease activity criteria, therapeutic options, medication dosing, monitoring recommendations, targets, definitions of a therapy failure and four therapeutic algorithms developed by a working group were agreed by web based survey to which all members of the GKJR have been invited. A final protocol with 4 consensus treatment plans (CTP) was agreed in a face-to-face consensus conferences employing modified nominal group technique.

**Results:**

The initial 17 definitions and recommendations for new-onset polyarticular JIA agreed by the working group reached >80% agreement in a web survey in 68 German paediatric rheumatologist. Four CTPs were developed based on treatment strategies for the first 12 months of therapy, as well as definitions for clinical and laboratory monitoring. The CTPs include a step-up plan (nonbiologic Disease-modifying antirheumatic drug [DMARD] followed by a biologic), a combination plan (combination of nonbiologic and biologic after failure of initial DMARD), an intensive pulse corticosteroid scheme in parallel with a DMARD followed by combination therapy and a multiple corticosteroids joint injections strategy in a treat to target approach. Step up will be guided by a treat to target strategy to reach a JADAS-improvement at month 3, acceptable disease at month 6 or 9 and JADAS remission or at least JADAS minimal disease activity at month 12.

**Conclusion:**

Standardized baseline work-up, disease activity evaluation and a definition of a treat to target approach will result in better health outcomes for polyarticular JIA patients. Four CTPs were developed for new-onset polyarticular JIA, which coupled with data collection at defined intervals will be evaluated and improved to optimize management of polyarticular JIA. Harmonization of treatment will be the basis for future comparative effectiveness research.

**Electronic supplementary material:**

The online version of this article (10.1186/s12969-017-0206-9) contains supplementary material, which is available to authorized users.

## Background

The outcomes of patients with polyarticuar juvenile idiopathic arthritis (JIA) have improved significantly due to the availability of more efficacious agntirheumatic therapies and improved treatment strategies [1–5]. Evidence-based guidelines for the treatment of polyarticular JIA exist in Germany but are limited in its scope [6]. More recently, treatment recommendations based on evidence and expert opinion for JIA patients have been developed by the American College of Rheumatology (ACR) in 2011 [7]. These have been updated for patients with sJIA in 2013 [8]. The North American Childhood Arthritis & Rheumatology Research Association (CARRA) has developed consensus treatment protocols (CTPs) based on the usual clinical practice of providers within CARRA [9, 10]. It has been added to the current knowledge, that an early response to therapy is related to a better outcome [11, 12].

The current situation in the care of rheumatic children and adolescents however is characterized by inadequate standardization and poor penetration of such therapies and recommendations in clinical practice. Consequently, children and adolescents with rheumatic diseases are frequently treated too late or inadequately, or not according to the current treatment options.

Guidelines are based on verifiable scientific findings at highest quality based on double-blind randomized trials [13–18]. Therapy recommendations aim at an optimal therapy of the diseased child. On the other hand, the implementation of guidelines and recommendations for therapy the availability and, in particular, the authorization situation of medicines must be considered.

A solution of this situation is (1) establishment of a network of children’s rheumatology centers in Germany, (2) the implementation of international diagnostic and therapeutic standards, (3) the creation of coordinated alternative therapy protocols with free choice by the treating physician, (4) comparative evaluation (5) the optimization of the therapeutic protocols by gradually improving the treatment protocols.

The Society for Childhood and Adolescence Rheumatology (Gesellschaft fuer Kinder- und Jugendrheumatologie, GKJR) has therefore set up a process guideline and has appointed a commission "Projects for the Classification, Monitoring and Therapy in Paediatric Rheumatology" (Projekte zur Klassifikation, Ueberwachung und Therapie in der Kinderrheumatologie, PRO-KIND), which coordinates various workgroups whose aim is harmonization and optimization of diagnostics, documentation and treatment of children and adolescents with rheumatic diseases. The working groups do not elaborate guidelines, but action protocols for everyday clinical practice. These are adapted for selected juvenile rheumatic diseases, based on the existing evidence on the use of therapeutics and the current practice in clinical practice, regarding diagnostics and follow-up documentation.

The protocols to be prepared are intended to serve as a guide to the indication, implementation and monitoring of the therapy in everyday clinical practice. The therapy should be directed towards the highest possible benefit for the patient, for example remission of the disease, as long as a definition for this is available.

## Methods

### Description of the process

The protocols for the classification, monitoring and therapy in pediatric rheumatology (PRO-KIND) - Polyarticular Juvenile Idiopathic Arthritis were followed by a step-by-step process of preparation of the consisting therapeutic protocols of the GKJR. First of all, all members of the GKJR were invited by e-mail to participate to a preparatory meeting on January 15, 2015 in Sankt Augustin. Here, twelve different project groups, ((1) polyarticular juvenile idiopathic arthritis (JIA), (2) persistent oligoarticular JIA, (3) Enthesitis-related Arthritis-JIA, (4) systemic JIA, (5) JIA-associated uveitis, (6) familial mediterranean fever, (7) hereditary autoinflammatory syndromes CAPS/TRAPS/HIDS, (8) systemic lupus erythematosus, (9) juvenile dermatomyositis, (10) non-bacterial osteitis, (11) juvenile psoriatic arthritis and (12) juvenile systemic vasculitis were formed. Secondly, on March 5th, 2015, all members of the GKJR were invited to participate actively. Each individual working group had one speaker and one coordinator who where requested to formally describe the voting process within the group.

Within the framework of the preparatory meeting, a possible procedure for the consensus of protocols by working groups and the GKJR was proposed. The presentation included a multi-stage approach with different consensus processes:

1. Creation of a “draft” by the working group
2. E-mailing to all participants of the working group
3. Consenting within the working group by means of multiple telephone conferences. A consensus level of at least 80% of the participants was mandatory.
4. Web-Survey 1: All members of the GKJR are asked to participate. A consensus of at least 80% of the participants was mandatory to reach consensus.
5. If necessary, repeated consenting within the working group for preparation of a second web-survey
6. If necessary a Web-Survey 2 inviting all members of the GKJR
7. Consenting and decision finding by a face-to-face meeting to which all GKJR members were invited
8. Authorization of the protocol by the board of the GKJR and consent to publication.

In order to participate in the project "Therapy Protocols of the GKJR - Polyarticular Juvenile Idiopathic Arthritis" presented here, 6 board-certified pediatric rheumatologist declared their willingness to participate in core working group. The further process consisted of several steps:

Consensus building within the working group regarding 17 recommendations over the course of 3 telephone conferences (on 26.5.15, 20.7.15, 28.7.15). The statements were related to the following topics: (1) definition of disease with criteria for diagnosis and subclassification of juvenile idiopathic arthritis, (2) basic diagnostics, (3) recording of disease activity, (4) prognostic parameters, (5) definition of safety parameters and intervals of analyses (6) therapeutic targets, (7) therapeutic regimens, and (8) four different therapeutic algorithms. During preparation of the different statements the current evidence record according to AHRQ (Agency for Healthcare Research and Quality), the drug licensing situation and corresponding literature were taken into account. A consensus was defined as being achieved if at least 80% agreement between the participants of the telephone conference was reached.

Four therapy algorithms were proposed by the core working group. Approval of the medication was taken into consideration as well as the therapeutic variability in routine care, evaluated by using data from the ICON cohort established from 2011 to 2013 [19]. According to the approval, the initial use of methotrexate as first disease modifying drug seemed obligatory. Corticosteroids were frequently used both systemically and or as intra-articular injection. Biologics are approved for therapy failure and can be used either in combination or as monotherapy, taking into account the approval. The selection of the biologic should be the responsibility of the treating physician with the exception that Abatacept is approved only as a second line biologic. Time lines for evaluation of treatment response and measures for assessment of the efficacy and / or the therapy failure were also proposed by the core working group.

The consensus reached within the working group was subsequently submitted to all members for consent. This was done by means of a web-based survey (Survey-Monkey), which was invited on 15.11.2015 and 6.12.2015 by the branch office. The survey was considered finished on 7.1.2016. A total of 68 out of 102 certified pediatric rheumatologists of the GKJR took part in the survey. The representative nature of the survey participants for the care situation in Germany was determined by a cross-comparison with the National Pediatric Rheumatological Database in Germany (core documentation).: In the core documentation, 58 institutions had documented a total of 6691 patients with JIA in 2014. Of these, 5085 (76%) patients were cared for by the physicians also participating in the web survey. From larger institutions, more than 1 physician participated in the survey. Physician level: 57 of the survey participants contributed also to the 2014 German core documentation. In addition, physicians from 11 institutions participated in the survey, whose patients were not documented in the core documentation in 2014.

In the context of the web survey, the level of agreement was determined for each of the 17 statements. Possible answers were either

- I agree.
- I agree with the essential statement. I would like to propose the change mentioned below.
- I can not agree with the statement without the change mentioned below.
- I can not agree with the statement.

The first two possible answers were formulated in such a way that consent was given without a condition. There was the possibility of submitting a proposal for change in the choice of the second answer. When the third or fourth answer was chosen, the statement was not approved, the third response making the consent dependent on a change in the statement. The selection of the first two possibilities of response was defined as consent, the selection of the last two answers was not interpreted as consent to the unchanged statement. Following the survey, GKJR members were invited to a face to face meeting for the final consensus process on the recommendations. A U-shaped table order was used. Discussion was led by an external pediatric rheumatologist who did not participate in the survey. Survey questions, answers and comments/suggestions were presented by one of the authors (AR). Every participant was asked to give his opinion. If voting on a change was requested, an 80% level of agreement was required for acceptance. In general, minor and editorial changes were agreed. The final therapeutic protocols were submitted to the GKJR Management Board and were accepted in 2016.

## Results

Results of the working group Polyarticular juvenile idiopathic arthritis are presented here. Seventeen Statements have been consented within the PROKIND-working group and were presented to member of the GKRJ via a web survey (Additional file 1: Table S1).

The results of the web survey were analyzed and a very high degree of approval was found. Overall, agreement was reached between 86.7% and 98.3%, which means that all statements were formally accepted (Additional file 1: Table S2). The table shows the approval rates of 68 participants of the web survey.

The results of the web survey were presented in a face to face consensus meeting on 9.3.2016 to which all members of the GKJR were invited.

Repeatedly suggested modifications from the web survey to the 17 statements were compiled and theses proposed changes were discussed and modifications were included in the final statements if 80% agreement could be reached.

### Diagnosis

The current diagnostic ILAR-criteria as modified 2001 [19] are to be used, until new criteria will be agreed on as proposed [20], including disease onset age, symptom start before the age of 16 years, presence of a confirmed arthritis (defined as inflammatory swelling and / or inflammatory painful restriction of movement) of at least 6 weeks duration, presence of cumulatively at least 5 affected joints (polyarthritis), exclusion of other causes for an arthritis and exclusion of other JIA categories (psoriatic arthritis, HLA-B27 in males >6 years of age, systemic signs of disease, persistent oligo-articular JIA, Enthesitis related arthritis). Cases with active chronic uveitis are excluded here but topic of a separate protocol.

### Diagnostic workup at baseline

Diagnostic workup at baseline should include detailed patient’s history including family history and vaccination status. Clinical examination has to include complete joint status and clinically internal findings. Laboratory examination include erythrocyte sedimentation rate (ESR), blood cell counts, white cell differentiation, CRP, ASAT, ALAT, GGT, creatinine, uric acid, LDH, Ca, AP, phosphate, urine status and serologic tests for Rheumatoid Factors (RF), cyclic -citrullinated peptides (CCP) -antibodies, ANA, HLA-B27, IgG, IgA, IgM as well as infectious diseases, Hepatitis B surface-antibody titer, Measles- antibody titer, Varicella-Zoster-virus- antibody titer if vaccination status is uncertain. Also a suitable test for the exclusion of an active or latent tuberculosis, for example, Quantiferon test or tuberculin skin test in children below 5 years of age, is recommended before starting treatment with a biologic agent.

### Evaluation of disease activity

Instruments to be used for evaluation of disease activity are active joint count, Childhood Health Assessment Questionnaire (CHAQ) [21]; pain measurement using a visual analogue scale (VAS) or a numeric rating scale (NRS), duration of morning stiffness and Juvenile Arthritis Disease Activity Score (JADAS) [22] with the four domains (1) Active joint count (swollen or tender joints with movement restriction), (2) Physician’s assessment of global disease activity (VAS or NRS), (3) Parent/Patient ‘s assessment of global disease activity (VAS or NRS), (4) ESR or CRP. The Juvenile arthritis disease activity score (JADAS) is recommend for the assessment and monitoring of disease activity as well as for the definition of a target to treat to as follows: JADAS 10 ≤ 1 defining JADAS remission, JADAS 10 ≤ 3.8 defining minimal disease activity and JADAS 10 ≤ 5.4 defining an acceptable disease activity from parents’ perspective [23].

Imaging assessments include sonography of affected or suspicious joints, X-ray of both hands and wrists especially in patients with RF + or CCP +, Magnet-resonance-imaging (MRI) if affection of temporomandibular joints, cervical spine or hip joints is suspected.

#### Prognostic parameters

A more favorable prognosis of the joint disease is presumably associated with the presence of ANA, the absence of RF and the absence of antibodies to CCP. Indicators of an adverse prognosis of the joint disease are the presence of RF and/or CCP-antibodies, the presence of arthritis of the hip joints, wrists, or temporomandibular joints and the presence of radiological damage, i.e. erosions or joint space narrowing.

#### General therapy guidelines

The pharmacotherapy of the JIA has changed significantly over the last 15 years. Although it initially consisted of varying combinations of non-steroidal anti-inflammatory drugs (NSAID), systemic and intra-articular corticosteroids and classical non-biologic disease modifying drugs (DMARDs), e,g. Methotrexate, an increasing number of biologic DMARDs has firmly established themselves (Table 1). Methotrexate is approved for treatment of polyarticular JIA patients at least in Germany. At present, European Medicines Agency (EMA) approvals are available for the treatment of polyarticular JIA for a total of 5 biologics, abatacept, adalimumab, etanercept, golimumab, and tocilizumab after having demonstrated their efficacy in the treatment of JIA by significantly increasing the success rate of reaching JIA ACR criteria or by avoiding disease relapses [13–18].

**Table 1:** Drug recommendation and dosing (Level of evidence grading according to Burns et al. [30])

Non-Steroidal-Anti-Inflammatory Drugs (NSAIDs)	No prolonged monotherapy with NSAIDs (without corticosteroid injections) in patients with active arthritis. NSAID monotherapy (without additional therapy) for longer than 2 months is unsuitable for patients with active arthritis, regardless of the presence of parameters indicating poor prognosis. Approval status and dosages have to be considered:Naproxene, 10–15,mg/kg bw, tablets approved from 12 years, juice formulation age limit >1 year. Ibuprofen, 30–40 mg/kg bw, approved from age 6 months. Indometacine, 2–3 mg/kg bw, approved from age 2 years, juice formulation available Diclofenac, 2–3 mg/kg bw, approved from age 9 years Meloxicam, 0,25–0,375 mg/kg bw, not approved or JIA, approved for Rheumatoid Arthritis, Ankylosing Spondylitis and arthrosis if age is >16 years Celecoxib, 6–12 mg/kg bw, not approved for JIA in Germany, approved in the USA for children 2 years of age
Systemic corticosteroids	Systemic high-dose corticosteroid therapy can be indicated in the presence of significant immobilizing disease activity. A systemic low-dose corticosteroid therapy can be used in long-term therapy, e.g. can be indicated in the presence of considerable morning stiffness. Intravenous high-dose steroid pulse therapy may be initially indicated for high disease activity, immobilizing disease and critical extra-articular manifestations. Short term highly dosed corticosteroids (considerable immobilizing disease activity) Prednisone, Prednisolone or Methylprednisolone at a dosage up to 2 mg/kg bw daily (up to 60 mg) in 3 ED for about 2 weeks, followed by tapering of the dosage about 25% per week over the next 4–8 weeks. Low dosed corticosteroids (in long-term therapy, for example, with severe morning stiffness) <0,15 mg/kg bw, probably alternate day <0,2 mg/kg bw every 48 h. Corticosteroid Pulse Therapy (with high disease activity, immobilizing disease, critical extra-articular manifestations) Methylprednisolone, 10-30 mg/kg (up to 1000) as infusion on 3 consecutive days. To be repeated after 2–4 weeks.
Intraarticular corticosteroids	Intraarticular corticosteroid therapy may be indicated for any joint with active arthritis. It may be used as an initial therapy, as a component or in addition to other therapies. Injections can be repeated in several months intervals; Triamcinolone hexacetonide (TH) is preferable to other preparations. TH 0.5-1 mg / kg bw can be used in large joints (knee, hip, shoulder), up to 0.5 mg / kg bw in medium-sized joints (hand jump, elbow joints) and up to 2 mg in small joints (finger or toe).
Methotrexate	The use of methotrexate has been justified by a double-blind placebo-controlled study of polyarticular JIA with evidence level 1A [13]. It is indicated for all patients if after the initial diagnostic phase active polyarthritis is present. Application can be orally or s.c. at a dosage of 10–20 mg / m2 / once per week. Additionally folic acid 5 mg, 1 / week, 24 h after methotrexate is optional.
Sulfasalazine	The use of sulfasalazine can be justified with evidence level 2 because of the results of a double-blind placebo-controlled study in polyarticular juvenile arthritis [14]. Is is recommended only for HLA-B27 positive patients, possibly indicated in combination with MTX. The target dosage of 30–50 mg/kg/day is stepwise reached by increase from 10 mg/kg over 2–4 weeks.
Hydroxychloroquine	The use of hydroxychloroquine can be justified on the results of a double-blind placebo-controlled study in polyarticular juvenile arthritis with evidence level 2 [15]. Dosage 5–7 mg/kg based on ideal weight It is not indicated as monotherapy and in exceptional cases in combination with methotrexate.
Leflunomide	The use of leflunomide can be justified with the evidence level 2 because of a double-blind, controlled study with inconclusive results in polyarticular juvenile arthritis [16]. Not approved for treatment of JIA, therefore not recommended for therapy. Dosage in children up to 20 kg body weight 10 mg daily, 20 mg kg body weight 15 mg daily and 20 mg in over 40 kg body weight.
Etanercept	The use of etanercept is justified by a double-blind, placebo-controlled study in polyarticular juvenile arthritis with the evidence level 1B [1]. Dosage 2 × 0.4 mg or 1 × 0.8 mg / week or 0.8 mg / kg / week in 1–2 injections with a maximum weekly dosage of 50 mg.
Adalimumab	The use of adalimumab can be justified by a double-blind, placebo-controlled study in polyarticular juvenile arthritis with the evidence level 1B [2]. Up to age of 13 years the dosage is 24 mg / m2 every 2 weeks, maximum 40 mg / injection, from 13 years on 40 mg / 2 weeks, in exceptional cases 40 mg / week.
Tocilizumab	The use of tocilizumab is justified by a double-blind, placebo-controlled study in polyarticular juvenile arthritis (level 1B) [3]. The dosage i.v. is 10 mg / kg / 4 weeks if body weight is <30 kg. and 8 mg / kg / 4 weeks if body weight ≥ 30 kg up to 800 mg / application.
Abatacept	The use of abatacept is justified by a double-blind, placebo-controlled study in polyarticular juvenile arthritis with evidence level 1 [4]. The drug is approved after failure of TNF inhibitors only. An iv dosage of 10 mg / kg at week 0, 2 and 4; then every 4 weeks is recommended.
Golimumab	The use of golimumab is justified with the evidence level 2 (15) due to a single inconclusive double-blind, placebo-controlled study in polyarticular juvenile arthritis (15). Dosage: 50 mg/m^2^ s.c. every 4 weeks for adolescents with polyarthritis with a body weight of 40 kg. Obligatory combination with methotrexate.

The present protocols of the GKJR are based on the results of controlled randomized trials and are taking drug approval into account to increase the feasibility of the recommendations. The final selection of the drug therapy is reserved for the treating physician in the sense of a preservation of his free choice of drugs for therapy, in particular the choice of a biologic agent. Both scientific knowledge about the efficacy in treatment of accompanying uveitis and the individual situation of the child, which favor a subcutaneous or intravenous administration of a drug, should be considered.

A recommendation for an “off-label” use is expressly not given. If a severe health impairment or pain related illness could not be treated effectively due to lack of therapeutic alternatives and positive adequate research results are available that the medicinal product could be applicable for the indication in question, an “off label” therapy may be indicated by the attending physician and is covered by the health insurance companies. (Federal Court decision Az: B 1 KR 37/00 R on March,19, 2002).

The indication for treatment, the exact choice of drug and the exact dosage of the medication lays in the full responsibility of the treating physician. The selection of drugs should take into account the efficacy, tolerability and long-term safety as well as the approval status. Whenever possible, drugs authorized for the specific indication, age and dosage are to be used. Differences in the expected efficacy should be respected, e.g. Adalimumab may be preferred over Etanercept in the presence of recurrent or chronic uveitis. The therapy during the diagnostic evaluation should be symptomatic. Prognostic parameters as well as validated criteria for assessing efficacy of treatment (eg. minimal ACR30, decrease of JADAS10, improvement of physician / patient global assessment) are to be considered for initiation, escalation and termination of therapy.

### Intervals for clinical control analyses, safety parameters and verification of improvement

Initial clinical control and laboratory safety assessment intervals every 4–6 weeks are recommended until an improvement has occurred. Thereafter every 3 months. Laboratory assessments should include: Blood count including differential blood counts, ESR, C-reactive protein (CRP), creatinine, ASAT, ALAT, GGT, LDH every 4–6 weeks until improvement has occurred, uric acid: this is carried out 1 / year, RF, if initially positive: 4/year if used as a parameter for efficacy.

### Definition of therapeutic targets

The aim of the therapy is to achieve remission - as measured by the definition according to JADAS (JADAS10 ≤ 1) or the provisional definition for clinical remission [23].

An acceptable response is the achievement of a condition of low or minimal disease activity (MDA), as defined by JADAS (JADAS10 ≤ 3.8) and a “Parent acceptable symptom state” (JADAS10 ≤ 5.4) and a child acceptable symptom state, respectively (JADAS10 ≤ 4.5) [23].

A minimally necessary response is to achieve an improvement as measured by a decrease in the JADAS 10 (definition of JADAS response [24] (Table 2).

**Table 2:** Cut off for improvement (according to ref. [16]). A minimal improvement can be assessed by either a decrease of the absolute JADAS or a relative decrease

	JADAS10 at Baseline		
	>5–15	>15–25	>25–40
Cut-off for improvement absolute ΔJADAS10	≥4	≥10	≥17
Cut-off for improvement relative ΔJADAS10	≥41%	≥53%	≥57%

In the case of already present structural changes, prevention or progression of joint damage is a therapeutic goal.

### Definition of a therapy failure and indication for a change of therapy

A failure of the therapeutic regimen and indication to change therapy for JIA is indicated by insufficient improvement, i.e. JADAS minimal improvement not reached (as defined in Table 2) after month 3 of treatment. Regardless of the JADAS value a necessary daily prednisolone equivalent dosage of ≥0.2 mg / kg to control disease activity later than month 3 also indicates treatment failure, as well as deterioration (re-increase) of the JADAS later than month 3. JADAS “parent acceptable disease activity” not reached at month 6 (JADAS10 ≤ 5.4) or treatment goal “inactive disease” (JADAS ≤ 1) or “low disease activity” (JADAS ≤3.8) not reached after 12 months of therapy are further criteria for treatment failure.

### Therapy algorithms

**Fig. 1:**
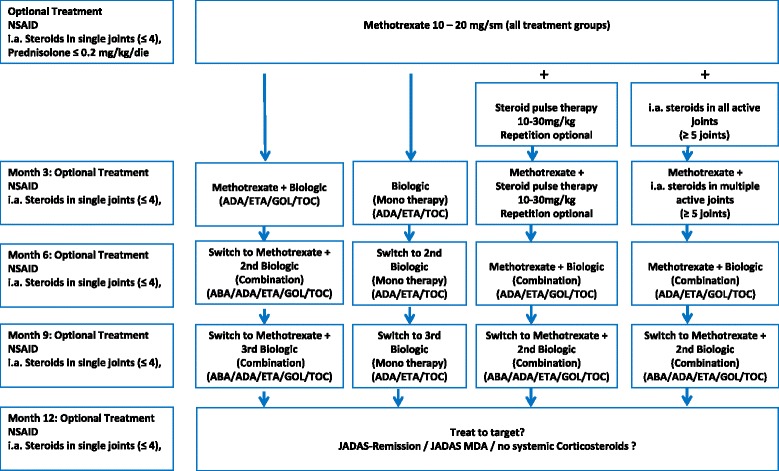
Therapeutic algorithm with 4 equally applicable consensus treatment plans. Initial treatment with methotrexate is intended for all patients with the diagnosis of polyarticular JIA. Non-steroidal-antiinflammatory drugs (NSAID) and up to 4 intraarticular joint injections with Triamcinolone hexacetonid are facultative on the discretion of the physician. Efficacy and tolerability should be evaluated every 3 months. The existing therapy will be continued if the therapeutic goals have been achieved, but should be altered if these have not been achieved. The treatment goals formulated for month 3, 6, 9 and 12 become more stringent with duration of therapy. The selection of the biologics is the responsibility of the treating physician. The approval for age and weight should be considered. ABA = abatacept, ADA = adalimumab, ETA = etanercept, GOL = golimumab, TOC = tocilizumab

The therapy algorithms represent possibilities for the succession of therapeutic decisions. Four different treatment algorithms have been accepted (Fig. 1). All algorithms have in common to treat all patients with active polyarticular JIA with Methotrexate and to provide concomitant treatment with NSAIDs, oral prednisolone (equivalent of 0.2 mg/kg/day) and intraarticular corticosteroids optionally. Algorithm No. 1 is characterized by an add-on therapy of Methotrexate with a biologic starting at month 3. Algorithm 2 is characterized by switching to a monotherapy with a biologic. Algorithm 3 is characterized by an initial intravenous steroid pulse therapy and algorithm 4 is characterized by an initial intraarticular steroid therapy into numerous, at least more than 4 active joints.

The choice of one of the algorithms and of individual drugs lies in the responsibility of the treating physician. The effectiveness of the therapy should be assessed on a regular basis every 3 months using the JADAS. If disease activity does not improve sufficiently modification of treatment is recommended according to one of the 4 algorithms outlined in Fig. 1. After starting therapy with Methotrexate, an assessment of the effectiveness every 3 months is recommended. The decision for therapy modification is to be made depending on the improvement of the JADAS, the achievement of a therapy goal taking into account the inflammatory activity (active joints).

After starting treatment with a biologic, assessment of the effectiveness and possibly modification of treatment every 3 months is recommended the same way. The treatment goals (JADAS improvement, JADAS defined minimal disease activity and JADAS-remission) should be aimed for.

So far, after reaching target of treatment at month 12 no advice for a treatment withdrawal is given. A following routine care is recommended.

## Discussion

With the constant development of new effective therapeutic agents for the treatment of JIA the goal of JIA treatment has changed over the last decades. Remission of disease or at least minimal disease activity can be reached in a high percentage of patients and should be the aim of any JIA treatment regime [25]. This aim can be reached with different treatments/treatment strategies. Registry and national core documentation data demonstrate a variety of treatment strategies are applied in clinical routine practice throughout Germany by the early use of Methotrexate as conventional DMARD and of biologics [26, 27]. Actual therapeutic reality however, also include high dose steroid pulses at baseline although the evidence for such a treatment is scarce as well as for multiple intraarticular joint injections.

Standardized approaches to diagnosis and treatment are transparent and are considered to help reaching the treatment target earlier and in a greater proportion of patients than routine unguided care.

With the assumption, that a strict treatment regime with regular critical evaluation of disease activity as the main indicator for effective treatment and resolute modification of treatment in case of inefficacy/insufficient efficacy within determined intervals is superior to individual treatment practices in general, this protocol has been developed. The treating physician/pediatric rheumatologist is not influenced in the choice of treatment, especially the choice of any of the approved biologics. Also effectiveness of different agents varies between patients. Heterogeneity of disease presentations and disease courses should be acknowledged.

A very important question in clinical practice is when to change treatment, if the patient shows insufficient treatment response. For different agents the time to onset of effect has to be taken into account. Over-treatment should be avoided. The measuring of treatment success poses also a challenge and different approaches have been chosen. While the CARRA protocols [10] use the physician global assessment, ability to taper/discontinue steroids as well as a not clearly defined “patient much improved” statement as criteria for treatment success, the 2013 updated ACR recommendations take into account prognostic factors as well as threefold classification of disease activity using the 4 parameters, that are also used in the JADAS. For the present protocol the JADAS as a validated, time-efficient, simple tool for evaluation of treatment efficacy was chosen [21].

On the international level, networks were established which, on the basis of therapy recommendations, produced protocols for a standardization of the therapy and, via outcome analyzes, allowed a comparative measurement of the therapeutic results and then allowed a stepwise improvement of the therapeutic recommendations [28]. The use of such internationally agreed protocols is limited in Germany as the insurance system covers approved treatment only and biologics in Germany are approved for refractory cases only. These protocols, including the here presented protocol all have in common a treat-to target approach with reassessment of the patient every few months and a stringent stepping-up of treatment, if the treatment response is inadequate. The ACR and CARRA protocols on polyarticular JIA are intended for patients with polyarticular ERA or psoriatic disease also. With different DMARD and biologic options, these subgroups will be addressed separately in the German protocols. The CARRA protocols propose 3 different treatment plans, two of them allowing treatment with biologic initially. Due to the approval situation in Germany, biologics can only be used after methotrexate therapy was unsuccessful or not tolerable. Further treatment approaches, which is not represented in either ACR or CARRA recommendations are scenarios 3 and 4 with either steroid pulse therapy or multiple joint injections. These approaches represent clinical practice in some German pediatric rheumatology centers.

The present protocol for the first year of treatment for polyarticular juvenile idiopathic arthritis is intended to serve as a guide to the diagnosis, clinical examination, indication, implementation and monitoring of therapy in everyday clinical practice. This standardized protocol is not intended to reflect each individual clinician’s usual practices, but to represent the general and most common approaches to treatment of pJIA by pediatric rheumatologists in Germany. The protocol is based on two statements of the German guideline for the therapy of juvenile idiopathic arthritis [6]: 1. The prerequisite for a successful therapy is an early diagnosis and allocation of the patients to specialists in pediatric rheumatology with competence and experience in the treatment of the JIA. 2. The objectives of the therapy are: the rapid and effective inflammation treatment with appropriate pain control, the control of the basic disease and possibly the remission induction, the avoidance of physical handicap by joint contractures, joint destruction, growth disturbance in the affected joints with the consequence of defects, preservation of the eyesight, avoidance of the impairment of internal organs, support for psychosocial stress on the patient and the family, ensuring a largely disorder-free somatic and psychosocial development of children and adolescents.

Criteria for which patient will be included/excluded, which baseline and follow up parameters should be evaluated and which response parameters can be used are commonly used and easily agreed on. The innovation of the project is the treat to target approach aiming to switch and modify treatment until the goal of improvement is reached. Furthermore, the target to treat to is stricter the longer the treatment duration. While at month 3, a measurable improvement of the JADAS is the first target to be reached by treatment, which has been defined earlier, at month 6, a maximum acceptable disease activity as defined by the JADAS is targeted. An unacceptable disease activity thus will lead to a change of the therapeutic regimen with the final target to reach being JADAS remission or at least JADAS defined low disease activity. This regimen will be tested during the first year of treatment, prospectively following patients newly diagnosed with pJIA. Participating centers will adhere to the strict regimen, while being independent in the choice of (b)DMARD. Ratios of patients reaching JADAS remission/MDA will be compared to those rates observed in cohort upon unguided treatment available via BIKER (Biologics in Pediatric Rheumatology Registry) and ICON data [27, 29].

These protocols are no guidelines but rather recommendations for diagnosis, treatment and surveillance. The aim is to standardize the care of children with polyarticular JIA aiming to measure, compare and finally improve the outcome of the disease. A future task will be the evaluation of the therapeutic results, which should lead to the optimization of the therapeutic protocols with the goal of a gradual improvement of the treatment protocols.

The establishment of a structure with a procedural regulation by the GKJR and the high level of willingness of the members of the GKJR for active cooperation and critical discussion represent a turning point for the future care of rheumatic children and adolescents.

Limitations of this protocol include that the recommendations proposed do not go beyond the initial 12 months; of course a long-term approach is envisaged with the target of remaining in a state of JADAS remission/MDA. The question of tapering or discontinuing medication due to remission of disease is not discussed. Newly arising/Prospective treatment options are not incorporated, but can be updated/added in the future.

## Conclusions

We established German Consensus Treatment Plans for children with new-onset polyarticular JIA. This collaborative effort of the investigators of GKJR should have a wide appeal and acceptability to pediatric rheumatologists across Germany and Austria. The next steps are to prospectively collect data on patients treated with these treatment plans as part of routine clinical care, and through an analytic process, identify the treatments with the best outcomes and least side effects for children with polyarticular JIA.

## Additional file

Additional file 1: Table S1. 17 Statements that were approved by the web-based survey with >80% approval. **Table S2.** Agreement of 68 participants of the web survey. The first two possible answers were formulated in such a way that consent was given without a condition. (DOCX 17 kb)

## Abbreviations

ACR: American College of Rheumatology
AHRQ: Agency for Healthcare Research and Quality
ALAT: Alanin-Aminotransferase
ANA: Antinuclear antobodies
AP: Alkaline phosphatase
ASAT: Aspartat-Aminotransferase
BIKER: Biologics in Pediatric Rheumatology Registry
Bw: Body weight
Ca: Calcium
CAPS: Cryopyrin associated periodic syndrome
CARRA: Childhood Arthritis & Rheumatology Research Association
CCP: Cyclic citrullinated peptide
CRP: C-reactive protein
CTP: Consensus treatment plans
DMARD: Disease modifying antirheumatic drug
EMA: European Medicines Agency
ERA: Enthesitis related arthritis
ESR: Erythocyte sedimentation rate
GGT: Gamma-Glutamyltransferase
GKRJ: German Society for Pediatric Rheumatology (Gesellschaft für Kinderrheumatologie)
HIDS: Hyper-IgD-Syndrome
HLA: Human Leukocyte Antigen
ICON: Inception Cohort of Newly Diagnosed Patients with Juvenile Idiopathic Arthritis
Ig: Immunoglobulin
JADAS: Juvenile Arthritis Disease Activity Score
JIA: Juvenile idiopathic Arthritis
Kg: Kilogram
LDH: Lactate dehydrogenase
MDA: Minimal Disease activity
NRS: Numeric rating scale
PRO-KIND: Protocols on classification, monitoring and therapy in children’s rheumatology
RF: Rheumatoid Factor
SHARE: Single Hub and Access point for paediatric Rheumatology in Europe
TRAPS: TNF-Receptor associated periodic syndrome
VAS: Visual analogue scale
