# Supplementary material for: Protocols on classification, monitoring and therapy in children’s rheumatology (PRO-KIND): results of the working group Polyarticular juvenile idiopathic arthritis
Source: Pediatr Rheumatol Online J. 2017 Nov 7;15:78. doi: 10.1186/s12969-017-0206-9 (PMC5678777; doi:10.1186/s12969-017-0206-9)
Supplement: Additional file 1: Table S1. — 17 Statements that were approved by the web-based survey with >80% approval. Table S2. Agreement of 68 participants of the web survey. The first two possible answers were formulated in such a way that consent was given without a condition. (DOCX 17 kb) [file 12969_2017_206_MOESM1_ESM.docx]

**Additional files**

Additional file 1: Table S1 17 Statements that were approved by the web-based survey with >80% approval.

| Question 1 | The consensus therapy protocols are intended for the following ILAR-defined JIA categories (Inclusion criteria): Polyarticular course, Rheumatoid factor positive and negative polyarthritis, Extended oligoathritis  The consensus therapy protocols should not apply to (exclusion criteria): any other JIA category (systemic JIA, psoriasis arthritis, ERA, persistent oligoarthritis) |
| --- | --- |
| Question 2 | The minimum required basic diagnostics procedures include: blood count including differential blood count, ESR, CRP, ASAT, ALAT, GGT, creatinine, uric acid, LDH, Ca, AP, phosphate, IgM rheumatoid factor, CCP antibody, HLAB27, ANA, IgG, IgA, IgM, Transglutaminase IgA antibodies. |
| Question 3 | Prior to immunosuppressive therapy vaccination antibodies (VZV, measles) should be measured if vaccination status is uncertain. Before start of a therapy with TNF inhibitors HBs-Antibody should be analyzed and a quantiferone or Elispot test to exclude TBC infection, respectively should be performed. |
| Question 4 | Sonographic examination at least of all clinically affected or suspected joints is recommended initially. MRI should be used if temporomandibular joints or cervical vertebral involvement is suspected. For children and adolescents with detectable rheumatoid factors and / or CCP antibodies X-ray examinations of the hands are recommended. |
| Question 5 | Assessment of disease activity and functional restriction should be performed using the Juvenile Arthritis Disease Activity Score (JADAS) and the Childhood Health Assessment Questionaire (CHAQ), respectively. The JADAS 10 of **≤**1 corresponds to a JADAS remission, a JADAS10 of ≤ 3.8 corresponds to JADAS minimal disease activity, a JADAS 10 of ≤ 5.4 corresponds to an acceptable disease activity. |
| Question 6 | Parameters for a doubtful or more serious prognosis may be: detectable rheumatoid factor, detectable CCP antibodies, affection of wrist, temporo-mandibular or hip joints, the presence of radiologically detectable joint damage |
| Question 7 | Definition of safety parameters for regular follow up examinations: blood count including differential blood count, ESR, CRP, Creatinine, ASAT, ALAT, GGT, LDH, uric acid, and rheumatoid factor if initially positive |
| Question 8 | Clinical control examinations should be carried out in intervals every 4-6 weeks until documentation of a satisfactory improvement, thereafter every 3 months. |
| Question 9 | Definition of the objectives of the pharmacoomedical therapy:: JADAS remission (JADAS10≤1) is the actual goal, JADAS-MDA (minimal disease activity, JADAS10 of ≤ 3.8) is an "acceptable" target, a JADAS10> 5.4 is an unacceptable disease activity. The Prevention of joint damage is a further therapeutic goal. |
| Question 10 | A symptomatic therapy with non-steroidal antiphlogistics should be performed in the presence of corresponding symptoms. Common dosages are given in table 2 |
| Question 11 | Therapy with corticosteroids can be indicated in certain situations. For example, see the following table   \|  \| Indication \| \| --- \| --- \| \| High dose,  prednisone, prednisolone or methylprednisolone in a dose of up to 2 mg / kg in 3 ED \| In the case of considerable immobilization \| \| low dose  <0.15 mg / kg, alternatively <0.2 mg / kg body weight every 48 h \| In long-term therapy, e.g. with considerable morning stiffness \| \| steroid pulse therapy  Methylprednisolone, dose: 10-30 mg / kg as intravenous infusion for 3 consecutive days \| Can be initially indicated in case of high disease activity, immobilizing disease, critical extra-articular manifestations \| \| Intraarticular corticosteroids \| Triamcinolone hexacetonide (TH) is preferable to other preparations  Dose for TH 0.5-1mg / kg in large joints (knee, hip, shoulder), up to 0.5 mg / kg in medium-sized joints (hand-, elbow joints) and up to 2 mg per small joint Fingers, toes). The therapy can be repeated in several months intervals. \| |
| Question 12 | The following are statements on the disease modifying therapy including a commonly used dosage (see table 2). |
| Question 13 | The following are statements on therapy with biologics including a commonly used dosages (see table 2). |
| Question 14 | 4 different therapy algorithms were presented. The selection of the therapy arm is the responsibility of the treating physician. The selection of each preparations is also the responsibility of the treating physician. The approval of the preparation shall be taken into account. In the presence of uveitis adalimumab should be considered preferable over etanercept. Therapeutic escalation is recommended in case of treatment failure such as a lack of an improvement of the JADAS. (see figure 1) |
| Question 15 | A therapy failure can be defined as the absence of an improvement in JADAS (according to Horneff & Becker, 2013, table 1). Independent of this, a non-tolerable steroid dose (≥ 0.2 mg / kg body weight per day) can also be defined as a therapy failure. |
| Question 16 | After a period of at least 6 months of therapy, a therapy failure can be defined as a JADAS score higher than that for acceptable disease activity (JADAS10 <5.4) |
| Question 17 | Furthermore, a therapy failure can be defined if after a period of therapy of at least 12 months minimal disease activity (JADAS10 <3.8) is not reached. |

**Additional file 2: Table S2** Agreement of 68 participants of the web survey. The first two possible answers were formulated in such a way that consent was given without a condition.

| **Statement** | **I agree** | **I agree** with the essential statement. I would like to propose a change | I cannot agree with the statement without a change | I can not agree with the statement | |
| --- | --- | --- | --- | --- | --- |
| **1** | 90.8% | 4.6% | 1.5% | | 3.1% |
| **2** | 50.0% | 40.6% | 6.3% | | 3.1% |
| **3** | 71.4% | 20.6% | 4.8% | | 3.2% |
| **4** | 59.4% | 26.6% | 7.8% | | 6.3% |
| **5** | 88.7% | 6.5% | 1.6% | | 3.2% |
| **6** | 77.8% | 15.9% | 6.3% | | 0.0% |
| **7** | 49.2% | 42.6% | 6.6% | | 1.6% |
| **8** | 85.5% | 12.9% | 0.0% | | 1.6% |
| **9** | 87.3% | 7.9% | 1.6% | | 3.2% |
| **10** | 79.0% | 19.4% | 1.6% | | 0.0% |
| **11** | 70.5% | 26.2% | 1.6% | | 1.6% |
| **12** | 72.6% | 19.4% | 6.5% | | 1.6% |
| **13** | 82.3% | 11.3% | 3.2% | | 3.2% |
| **14** | 67.8% | 20.3% | 10.2% | | 1.7% |
| **15** | 87.1% | 11.3% | 0.0% | | 1.6% |
| **16** | 90.3% | 6.5% | 1.6% | | 1.6% |
| **17** | 85.2% | 9.8% | 0.0% | | 4.9% |
